# Live imaging of intra-lysosome pH in cell lines and primary neuronal culture using a novel genetically encoded biosensor

**DOI:** 10.1080/15548627.2020.1771858

**Published:** 2020-06-09

**Authors:** Amy H. Ponsford, Thomas A. Ryan, Andrea Raimondi, Emanuele Cocucci, Susanne A. Wycislo, Florian Fröhlich, Laura E. Swan, Massimiliano Stagi

**Affiliations:** a Department of Cellular and Molecular Physiology, Institute of Translational Medicine, University of Liverpool, Liverpool, UK; b Experimental Imaging Center, San Raffaele Scientific Institute, Milan, Italy; c Division of Pharmaceutics and Pharmacology, College of Pharmacy and the Comprehensive Cancer Center, The Ohio State University, Columbus, OH, USA; d Department of Biology/Chemistry, Molecular Membrane Biology Group, University of Osnabrück, Osnabrück, Germany; e Centre of Cellular Nanoanalytics (CellNanOs), University of Osnabrück, Osnabrück, Osnabrück, Germany

**Keywords:** Chloroquine, fluorescence microscopy, lysosomes, MTOR protein, pH, V-type ATPase

## Abstract

Disorders of lysosomal physiology have increasingly been found to underlie the pathology of a rapidly growing cast of neurodevelopmental disorders and sporadic diseases of aging. One cardinal aspect of lysosomal (dys)function is lysosomal acidification in which defects trigger lysosomal stress signaling and defects in proteolytic capacity. We have developed a genetically encoded ratiometric probe to measure lysosomal pH coupled with a purification tag to efficiently purify lysosomes for both proteomic and *in vitro* evaluation of their function. Using our probe, we showed that lysosomal pH is remarkably stable over a period of days in a variety of cell types. Additionally, this probe can be used to determine that lysosomal stress signaling *via* TFEB is uncoupled from gross changes in lysosomal pH. Finally, we demonstrated that while overexpression of ARL8B GTPase causes striking alkalinization of peripheral lysosomes in HEK293 T cells, peripheral lysosomes *per se* are no less acidic than juxtanuclear lysosomes in our cell lines.

**Abbreviations:** ARL8B: ADP ribosylation factor like GTPase 8B; ATP: adenosine triphosphate; ATP5F1B/ATPB: ATP synthase F1 subunit beta; ATP6V1A: ATPase H+ transporting V1 subunit A; Baf: bafilomycin A_1_; BLOC-1: biogenesis of lysosome-related organelles complex 1; BSA: bovine serum albumin; Cos7: African green monkey kidney fibroblast-like cell line; CQ: chloroquine; CTSB: cathepsin B; CYCS: cytochrome c, somatic; DAPI: 4′,6-diamidino −2- phenylindole; DIC: differential interference contrast; DIV: days *in vitro*; DMEM: Dulbecco′s modified Eagle′s medium;‎ E8: embryonic day 8; EEA1: early endosome antigen 1; EGTA: ethylene glycol-bis(β-aminoethyl ether)-N,N,N′,N′-tetraacetic acid; ER: endoplasmic reticulum; FBS: fetal bovine serum; FITC: fluorescein isothiocyanate; GABARAPL2: GABA type A receptor associated protein like 2; GAPDH: glyceraldehyde-3-phosphate dehydrogenase; GOLGA2/GM130: golgin A2; GTP: guanosine triphosphate; HEK293T: human embryonic kidney 293 cells, that expresses a mutant version of the SV40 large T antigen; HeLa: Henrietta Lacks-derived cell; HEPES: 4-(2-hydroxyethyl)-1-piperazineethanesulfonic acid; HRP: horseradish peroxidase; IGF2R/ciM6PR: insulin like growth factor 2 receptor; LAMP1/2: lysosomal associated membrane protein 1/2; LMAN2/VIP36: lectin, mannose binding 2; MAP1LC3/LC3: microtubule-associated protein 1 light chain 3; MTORC1: mechanistic target of rapamycin kinase complex 1; PCR: polymerase chain reaction; PDL: poly-d-lysine; *PGK1p*: promotor from human phosphoglycerate kinase 1; PIKFYVE: phosphoinositide kinase, FYVE-type zinc finger containing; PPT1/CLN1: palmitoyl-protein thioesterase 1; RPS6KB1/p70: ribosomal protein S6 kinase B1; STAT3: signal transducer and activator of transcription 3; TAX1BP1: Tax1 binding protein 1; TFEB: transcription factor EB; TGN: trans-Golgi network; TGOLN2/TGN46: trans-Golgi network protein 2; TIRF: total internal reflection fluorescence; TMEM106B: transmembrane protein 106B; TOR: target of rapamycin; TRPM2: transient receptor potential cation channel subfamily M member 2; V-ATPase: vacuolar-type proton-translocating ATPase; VPS35: VPS35 retromer complex component.

## Introduction

Lysosomal signaling and lysosomal integrity have, for the past decade, been closely investigated as a likely primary pathological cause in numerous human disorders ranging from congenital disorders of lysosome-triggered transcription pathways [1] and cystic fibrosis [2] to risk factors associated with dementia [3–5], insulin signaling [6] and cancers [7,8].

In recent years, the lysosomal vacuolar-type H+-translocating ATPase (V-ATPase) has been found to be a key element of lysosomal nutrient sensing through MTORC1 in both yeast and mammals [9] with consequences for cellular aging and organismal longevity. The cytosolic component (V_1_ complex) of the V-ATPase is regulated and recruited by numerous lysosomal stressors, and the assembled V-ATPase itself is required to conduct amino acid sensing to the MTORC1 complex [9]. However, this is apparently uncoupled to lysosomal bulk pH and, thus, to degradative capacity.

Lysosomal positioning and transport have been linked to both lysosomal acidification [10] and MTORC1 signaling [11,12], suggesting that lysosomes can shift physiological and signaling properties over time. Treatments that alkalinize the lysosomal compartment are also modulators of lysosomal MTORC1 signaling [13]. However, re-acidification of this compartment does not reverse TOR signaling once induced [14]. This suggests that either these are distinct events of the lysosomal signaling cascade, or that after activation, lysosomal alkalinization is not necessary to maintain MTORC1 signaling. Thus, there is a need for an imaging tool that reports lysosomal pH over long periods of time with an unbiased selection of lysosomal compartments. Ideally, this tool should also allow for the purification of lysosomes for proteomics and *ex-vivo* manipulation of purified lysosomes to provide for matched *in vivo* and purified samples.

Currently, defects in lysosomal pH are most commonly revealed by the addition of diffusible dyes recruited to several organelles, such as LysoTracker, cathepsin substrates and acridine orange [5]. In these cases, the effects of dye concentration/recruitment, number of organelles per cell and pH are difficult, if not impossible, to deconvolve [15]. Lysosomal pH is also measured by ratiometric experiments where pulse-chased fluid phase markers [10], ratiometric pairs of two different lysosomal resident proteins [16], and ratiometric receptor ligands [17,18] have been used to label lysosomes, as well as pulse-chase experiments using pH-tuned nanoparticles, *e.g*. [19]. These latter approaches are limited because they can only allow measurements in a limited time window as pulse-chased probes are washed through the exo-endocytic system and experiments may suffer from incomplete penetrance of dye-loaded organelles. Pulse-chase-loaded fluid-phase dyes do not always completely access the peripheral pool of lysosomes even after overnight dye loading [10]. These older peripheral lysosomes may have different properties than newly labeled lysosomes.

To avoid these limitations in dye distribution, exogenous loading, and to allow long-term lysosomal pH monitoring, we built a genetically encoded sensor, which would allow us to stably express a fluorescence-based probe that constitutively resides in the lysosomes to monitor luminal pH over long periods of time. We, therefore, built a sensor based on ratiometric fluorophore fusions to endogenous lysosomal proteins. LAMP1 was chosen for several reasons: first, it is one of the most abundant lysosomal proteins, and is likely to be trafficked similarly in all cell types, and secondly, experiments had shown that its overexpression had minimal effects on lysosomal size, positioning or physiology [5]. It also is part of the lysosomal glycocalyx, which helps to shield the probe from proteolytic degradation. Of the two best-characterized members of the LAMP protein family, LAMP2 has a significant role in lysosomal cholesterol trafficking [20,21], making LAMP1 chimeras a better choice for an inert sensor of lysosomal physiology.

Using this reporter in both transiently and stably expressing cell lines, we found that lysosomal pH is remarkably stable over the whole cell cycle. Lysosomal pH was not influenced by the relative positions of the organelles in the cell. We confirmed that overexpression of ARL8B causes a striking alkalinization of peripheral lysosomes, while we demonstrated that MTORC1-induced TFEB translocation does not require lysosomal acidification, though many TFEB translocation-inducing treatments did result, as expected, in alkalinization of lysosomal organelles. We further demonstrated that this probe could be used to isolate and manipulate active lysosomes for on-bead experiments and mass-spectroscopy-based lysosomal proteomics. We present this probe as a useful tool to deconvolve aspects of lysosomal signaling and pH in physiological and pathological states in both *in vivo* and *in vitro* applications.

## Results

### A ratiometric LAMP1 fusion is stably incorporated in lysosomal membranes

**Figure 1.**
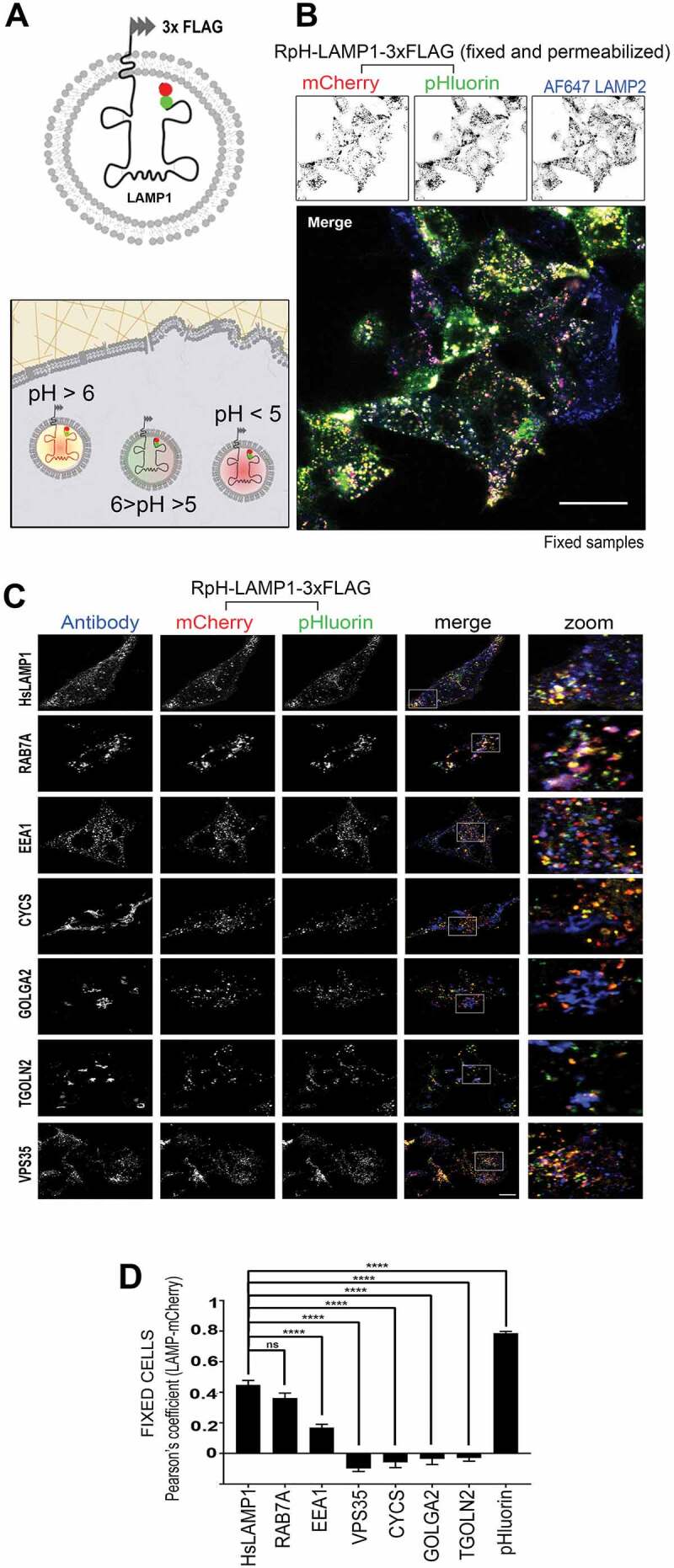
Design and expression of RpH-LAMP1-3xFLAG. (A) Topology of the probe with luminal Cherry-pHluorin tandem and cytosolic 3xFLAG tag. (B) A mixed culture of lentivirally transduced RpH-LAMP1-3xFLAG HEK293 T and untransduced HEK293 T cells showed colocalization of the probe with endogenous LAMP2 immunoreactivity. Fixation and permeabilization revealed that all mCherry-positive lysosomes were also positive for pHluorin fluorescence and LAMP2 immunoreactivity, indicating that the fluorophores were not degraded or quenched over time. Scale in B: 20 μm. (C) Colocalization of RpH-LAMP1-3xFLAG (red, green) with endogenous proteins (Alexa Fluor 647 fluorescence, blue) in stably transfected HEK293 T cells. Scale in C: 10 μm. (D) Quantification of colocalization of immunofluorescence signal with the mCherry channel. Colocalization was measured and then compared to 200 image randomizations to extract a Pearson’s coefficient (n = 30–40 cells per antibody). The mouse LAMP1 (mLAMP1) probe correlated well with itself (pHluorin channel), with endogenous HsLAMP1, RAB7A and to a lesser degree, the endosomal marker EEA1

To reliably measure lysosomal pH *via* ratiometric measurements, we made a tandem fusion of a pHluorin-mCherry linked to the luminal domain of the canonical marker protein of the lysosome, mouse LAMP1, with a cytosolic 3XFLAG tag which we called **R**atiometric **pH**luorin (RpH)-LAMP1-3xFLAG (Figure 1A**, S1A and S1B**). By using LAMP1, we were able to traffic our sensor to all lysosomes in the cell, without biasing our measurements to earlier or later lysosomes using pulse-chase application of dyes/ligands. The use of a genetically encoded ratiometric construct attached to a well-tolerated marker of the lysosome also meant that, unlike pulse-chase experiments, our design is compatible with live imaging over time courses appropriate for pharmacological experiments. This allows an accurate measurement of pH over extended time periods and allows correlation of lysosomal behaviors with a treatment history of a period of hours or days in culture.

Overexpressed RpH-LAMP1-3xFLAG incorporated efficiently in lysosomal organelles, and strongly co-localized with the endogenous pool of another member of the same family of lysosomal proteins, such as LAMP2 (Figure 1B). In these fixed, permeabilized cells, we also saw excellent correlation of mCherry and the unquenched pHluorin signal, showing that the fluorophore tandem is present in all mCherry-positive lysosomal structures.

To determine if our probe accurately recapitulated the trafficking of the endogenous LAMP1 protein, we made systematic studies in a HEK293 T line stably expressing our probe by determining colocalization with endogenous markers of subcellular organelles (Figure 1C). We then quantified the colocalization of the antibody stain with the mCherry channel of our probe using Costes method [22] and expressed this as a Pearson’s coefficient (Figure 1D). As expected, most of the probe co-localized with endogenous (human) LAMP1 and RAB7A, while a smaller fraction associated with the endosomal marker EEA1. As a control for the maximum possible colocalization, we showed that mCherry fluorescence correlated most strongly with itself (the probe’s pHluorin fluorescence) in fixed cells. No mCherry fluorescence was associated with retromer (VPS35), mitochondria (CYCS) or *cis-* or *trans*-Golgi (GOLGA2/GM130 and TGOLN2/TGN46, respectively), showing that this probe traffics to lysosomes normally.

### Monitoring lysosomal pH in heterologous cells and neurons

**Figure 2.**
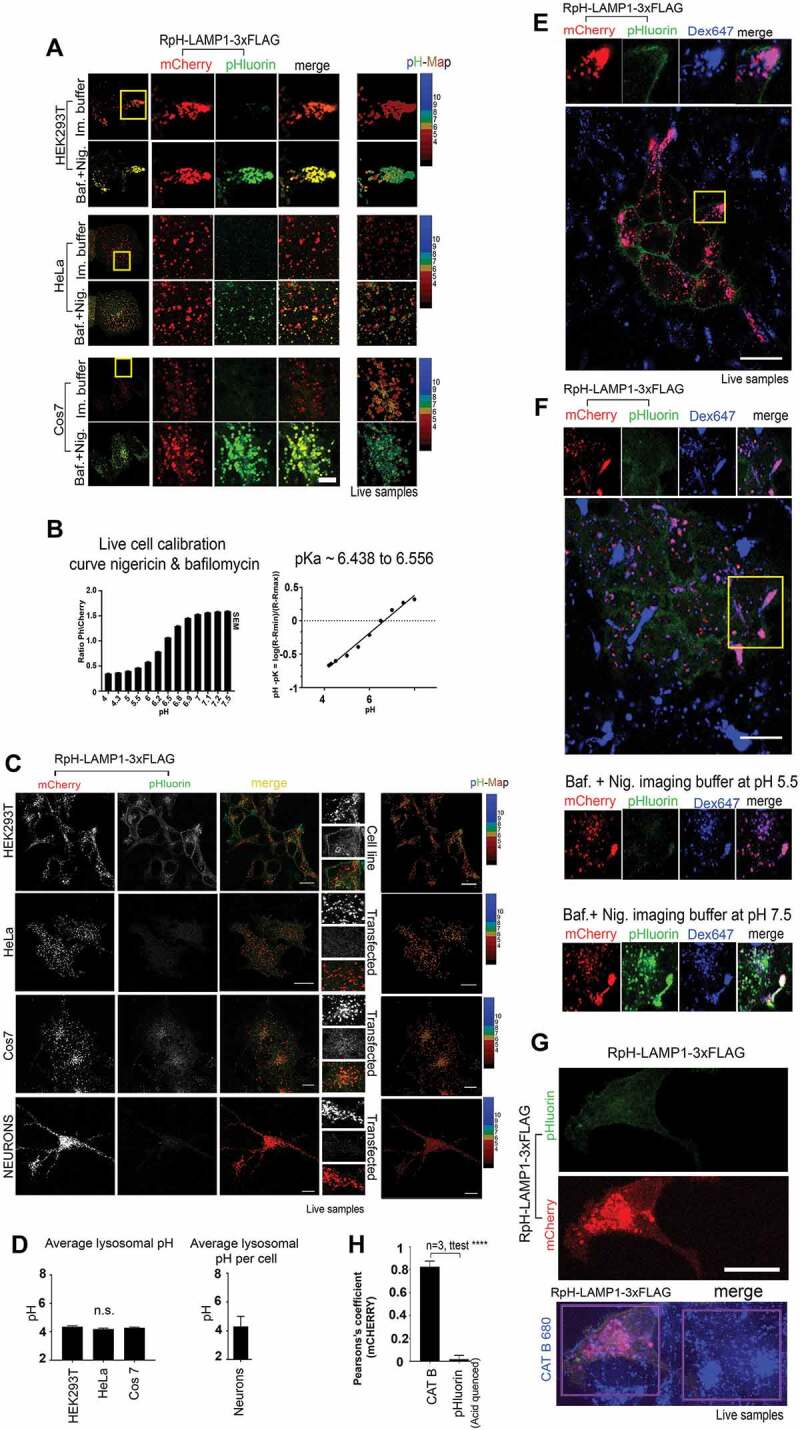
RpH-LAMP1-3xFLAG revealed stably acidic lysosomal pH in live-cell imaging. (A) RpH-LAMP1-3xFLAG expressed in HEK293T, HeLa and Cos7 cells showed the probe was found in acidic organelles (i.e., pHluorin was quenched). These organelles were unquenched in the presence of nigericin plus bafilomycin A_1_ (baf) in imaging buffer. Scale bar: 5 μm. (B) Calibration of RpH-LAMP1-3xFLAG in live-imaged HeLa cells *left panel*: ratio of pHluorin to mCherry fluorescence in nigericin plus baf-treated live cells with external buffers set at the indicated pH. *Right panel*: calculation of the pKa based on the experimental data. (C) Expression of RpH-LAMP1-3xFLAG in live imaging in HEK293T, Hela, Cos7 and primary chick neurons showing mCherry and pHluorin channels, merged image and calculated pH for mCherry-positive lysosomes. *Inset*: region of interest showing individual lysosomes. Scale Bar: 20 μm. (D) *Left panel*: pH calculation for cell lines per mCherry-positive lysosomes pH 4.35 ± 0.07 (HEK293T, n = 84), 4.2 ± 0.05 (HeLa, n = 183), 4.28 ± 0.05 (Cos7, n = 102), 6–8 independent fields of view per cell type. There was no significant difference in pH of mCherry-positive organelles between these cell lines (ANOVA) *Right panel*: average lysosomal pH as measured over all mCherry-positive fluorescence per cell, in chick primary neurons was pH 4.3 ± 0.7 (± StDev, n = 4). (E) Mixed HEK293T RpH-LAMP1-3xFLAG and untransduced co-cultures, live-imaged after overnight pulse and 3 h chase with Alexa Fluor 647 dextran to label total lysosomes. In live cells, pHlourin was quenched, indicating acid pH. Scale bar: 20 μm. (F) Permeabilization of dextran-labeled cells with 10 μM nigericin and blocking V-ATPase with 100 nM baf showed that RpH-LAMP1-3xFLAG HEK293T in dextran-labeled peripheral lysosomes responded to external buffer pH. *upper panel* before nigericin plus baf treatment, *middle panel* pH 5.5 *bottom panel* pH 7.5. Scale bar: 20 µm. (G) Live imaging of cells labeled overnight with a fluorogenic CTSB substrate (blue) in HEK293T RpH-LAMP1-3xFLAG and normal co-cultures showed CTSB activity was normal in cells stably expressing our probe. Scale bar: 20 µm. (H) In live cells, quantification of the correlation of CTSB substrate with the mCherry channel was high, whereas correlation of mCherry with pHluorin fluorescence in acid organelles was low

We then characterized our probe as a lysosomal pH sensor in living cells. We examined three standard cell culture lines (HEK293 T, HeLa and Cos7), Figure 2A. All three cell lines showed that lysosomes were acidic (pHluorin fluorescence was quenched) and that when the lysosomal V-ATPase was inhibited and permeabilized in living cells (bafilomycin A_1_ [baf] plus nigericin treatment) to allow infiltration of imaging buffer (pH 7.4), mCherry fluorescent lysosomes unquenched to reveal pHluorin fluorescence.

We calculated a pH calibration curve for the probe by live-cell imaging in bafilomycin A_1_ plus nigericin-permeabilized cells and recorded the ratio of pHluorin fluorescence to mCherry fluorescence for all mCherry-positive organelles (see calibration in Figure 2B **and Movie S1**, workflow presented in **Fig. S1C**). For each cell line, we established optimal ratiometric imaging conditions and calibrated them separately. We imaged both mCherry and pHluorin fluorescence simultaneously to minimize mismatch between the two imaging channels and adjusted imaging settings so that the two channels had the greatest dynamic range. We also collected calibration curves in fixed, permeabilized cells exposed to defined-pH buffers (**Fig. S2**). To calculate lysosomal pH, we calculated the ratio of pHluorin to mCherry fluorescence in objects defined as lysosomes by their fluorescence in the mCherry channel and calculated pH value from our calibration curve. We found that this sensor can reliably detect pH in the range pH 4.2-pH 7.1. Fitting our live-imaging data, we found that the pKa of this probe in live cells was approximately pH 6.5 (Figure 2B), close to the pKa of engineered exogenous ratiometric probes and of FITC dye (pKa 6.4 [10]), and better than that of the cytosolic mCherry-pHluorin tandem fusion, which has a reported pKa of 7.1 [23].

Having verified that our probe was functional, we then examined different cell types to see if lysosomal properties of standard cell-culture lines differed greatly (Figure 2C). To that end, we expressed our probe in stably transduced HEK293T cells, and transiently transfected HeLa cells, Cos7 cells and primary chick neurons. We performed calibrations using nigericin plus bafilomycin A_1_ for each cell line (as described above) and then measured average lysosomal pH in each cell line.

We found that the pH of lysosomes was highly consistent between all cell lines (Figure 2D). Average pH (mean ± SEM) per mCherry-positive lysosome in HEK293 T, HeLa, Cos7 and neurons was: pH 4.35 ± 0.07 (HEK293 T, n = 84), 4.2 ± 0.05 (HeLa, n = 183), 4.28 ± 0.05 (Cos7, n = 102). For each cell type, we measured over 6–8 independent fields of view. In chick primary neurons, due to closely packed mCherry-positive lysosomes at the cell center, we were unable to measure individual lysosomes and instead measured pH for all mCherry-positive fluorescence per cell. We found that the neuronal lysosomal pH was 4.3 ± 0.7 (± StDev, n = 4).

We compared our ratiometric probe to standard ratiometric dextran-based measurements of pH in HEK293T cells. First, to determine if lysosomal pH-sensing with our probe was changed by the addition of dextran, we labeled RpH-LAMP1-3xFLAG-expressing HEK293 T cells with an overnight pulse of Alexa Fluor 647-dextran. Using the pHluorin/Alexa Fluor 647-dextran pair to calculate pH (**Fig. S2D**, quantifications in **Fig. S2E**), we determined that the lysosomal pH was 4.09 ± 0.35 (n = 50), which agrees well with our measurements of lysosomal pH using the ratiometric Cherry-pHluorin pair. To confirm that overexpression of the ratiometric sensor does not change lysosomal pH as measured by dextran dyes, we took unlabeled HEK293 T and incubated overnight with a mix of FITC-, rhodamine- and Alexa Fluor 647-labeled dextrans. Using Alexa Fluor 647-dextran labeling as the reference channel as above, lysosomal pH was quantified as 4.1 ± 0.3 (n = 50) using FITC/Alexa Fluor 647 dextran pair, unchanged from the pH of RpH-LAMP1-3xFLAG-expressing HEK293 T. Using the more commonly used FITC/Rhodamine B pair, average lysosomal pH was found to be 4.05 ± 0.3 (n = 50). This result confirmed that our probe measured lysosomal pH as accurately as standard dye-based methods, and that its overexpression did not change lysosomal pH.

We then checked how well our lysosomal probe correlated with other live-imaging markers of lysosomal trafficking and function. Using overnight pulse and 3 h chase of Alexa Fluor 647-dextran, which fills the endolysosomal compartment on co-cultures of stably transduced and untransduced HEK293T cells, we saw that organelles labeled by RpH-LAMP1-3xFLAG took up external dye, as do surrounding non-clonal cells (Figure 2E). In these dextran-positive compartments, pHluorin fluorescence was quenched, indicating an acidic pH. When treated with the V-ATPase inhibitor 100 nM bafilomycin A_1_ and cell membranes permeabilized by 10 μM nigericin (Figure 2F), dextran-labeled organelles revealed pHluorin fluorescence in proportion to the pH set by external imaging buffers.

We also tested functional markers of lysosomes by examining the activity of an endogenous lysosomal hydrolase. We incubated cells with a non-fluorescent substrate (CATB 680) activated by cleavage by endogenous CTSB (cathepsin B; pH optimum 4.5 to 5.5) to produce an infrared fluorescence product at sites of CTSB cleavage (Figure 2G), commonly used as a marker of functionally active lysosomes. RpH-LAMP1-3xFLAG colocalized with CTSB hydrolase activity (colocalization quantified Figure 2H). As a control, we demonstrated that in live cells, there was no significant colocalization of mCherry with pHluorin fluorescence, showing that mCherry-positive organelles harboring cleaved CTSB substrate were acidic (i.e., pHluorin is quenched).

Long-term imaging (**Movie S2, Fig. S3A**, and pH calculation in **Fig. S3B**) showed that cells expressing our probe accumulated the reporter for active CTSB and continue to grow and migrate. Of interest, we noted that the addition of the weak base drug chloroquine (CQ, described below) causes the lysosome, as expected, to neutralize (increase in pHluorin channel), while the cathepsin cleavage product is retained in lysosomes, highlighting that cathepsin cleavage substrates indicate the historical sites of cathepsin cleavage, rather than reporting on the current pH of the organelle.

We also investigated whether this probe would identify lysosomal fusion events with the plasma membrane. While under our ratiometric imaging conditions, using a confocal microscope, we did not identify fusion events; however, we did find a small number of events by TIRF microscopy in transfected HeLa cells (**Fig. S3C**, gallery in **Fig. S3D and Movie S3**), which were enhanced, as predicted [24] by the addition of 300 nM thapsigargin, which releases Ca^2+^ to the cytosol from ER stores to trigger fusion of LAMP1-positive organelles with the plasma membrane [25]. Competence for lysosomal exocytosis differed among the tested cell lines. We observed occasional spontaneous fusion events in Cos7 (**Fig. S3E and Movie S4**), which became neutral on fusing with the plasma membrane (**Fig. S3F and Movie S4**), whereas in HEK293 T, TIRF imaging showed very few lysosomal vesicles fusing with the cell surface during our imaging period but a larger proportion of our probe’s green fluorescence spread out on the plasma membrane (**Fig. S3G and Movie S5**).

LAMP1, like many other type 1 transmembrane proteins, can reach lysosomes by a direct sorting route or traveling *via* the plasma membrane [26,27]. As a consequence, between 2–5% of LAMP1 is endogenously present on the cell surface, depending on the cell line in question [28–31]. The plasma membrane-localized pHluorin fluorescence in HEK293T stable lines was not changed by fixation (**Fig. S4A**), but when we adjust our imaging parameters in living cells so that the mCherry channel signal is saturated in lysosomes, mCherry fluorescence was also revealed on the plasma membrane (**Fig. S4B**), demonstrating that the plasma membrane fraction of RpH-LAMP1-3xFLAG was a minor fraction of total probe in these cells. Using an HsLAMP1 antibody, we also found a small fraction of LAMP1 was endogenously present on the cell surface in HEK293 T cells (**FigureS4C and S4D**).

### Lysosomal pH is stable during the cell cycle and cell migration

**Figure 3.**
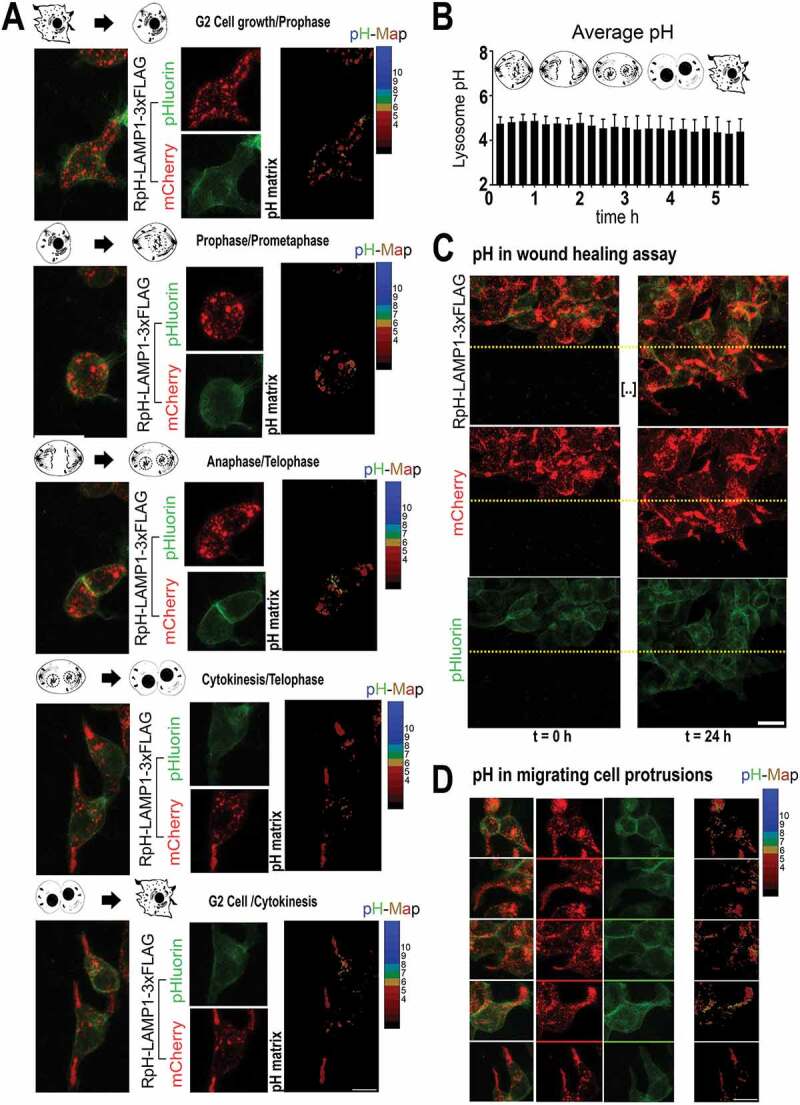
Lysosomal pH remained stable through cell cycle and cell migration. (A) RpH-LAMP1-3xFLAG in HEK293T cells undergoing spontaneous cell division (see **Movie S6**). Maximum projection of an image stack per frame, and pH calculation of mCherry-positive puncta in maximum projection image. Scale bar:10 µm (B) Average lysosomal pH per cell over the cell cycle. Four spontaneously dividing cells were measured and manually synchronized such that t = 0 was prophase (completely spherical cells) and followed by images of stacks every 15 min for 5 h 30 min thereafter. Data presented as mean ± StDev. (C) Maximum projection of image stacks in HEK293T cells migrating in a wound-healing assay. Lysosomal pH remained acidic throughout the cell culture for the entire live imaging period. Scale bar:10 µm. (D) Maximum projections of image stacks in migrating HEK293T cells in a wound-healing assay. Scale bar:10 µm

We next turned to examine the behavior of lysosomes in cells asynchronously undergoing cell cycle. HEK293T cells stably expressing RpH-LAMP1-3xFLAG were imaged for 48 h in DMEM with 10% FBS on a Zeiss LSM800 in an environmental chamber with 5% CO_2_ at 37°C, acquiring a stack of images every 15 min. Spontaneous cell divisions were observed (Figure 3A) and quantified for four cells. Average lysosomal pH was stable in cells throughout cell division (see Figure 3B **and Movie S6**), where cell divisions were manually synchronized such that t = 0 is prophase (completely spherical cells) and followed for 5 h 30 min thereafter.

We also measured the behavior of lysosomes migrating in wound-healing assays monitored for 24 h at 15 min intervals (**Movie S7**) after scrape injury (Figure 3C, timepoints t = 0 h and t = 24 h). By 24 h, we noted migrating HEK293T protrusions had a very strong accumulation of peripheral lysosomes, but these peripheral lysosomes remained acidic (Figure 3D).

### Lysosomal localization has no strong effect on pH in neurons and heterologous cells

**Figure 4.**
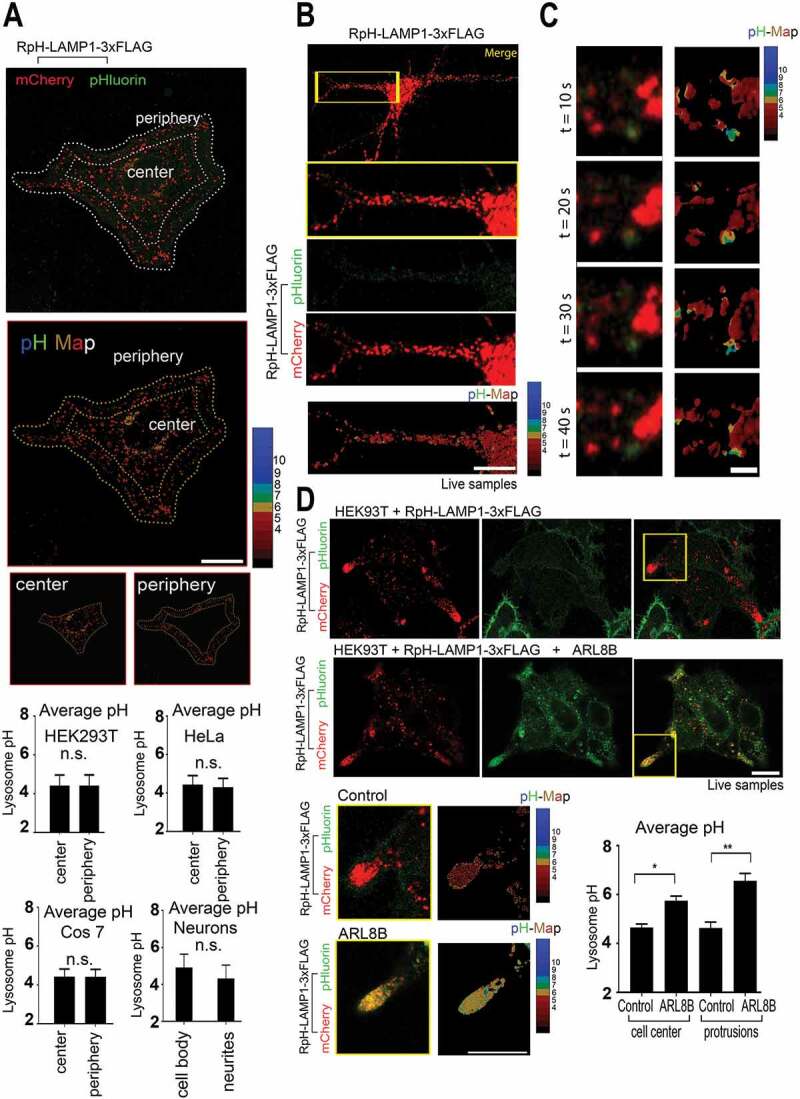
ARL8B overexpression markedly alkalinized lysosomes, but peripheral localization did not confer a similar effect on pH. (A) Peripheral vs central *puncta* quantified for HEK293T, HeLa, Cos7 and neurons n = 10 (cells per quantification), n = 4 (neurons), *n.s*.: not significant, ANOVA. pH of mCherry-positive organelles calculated from single confocal planes of live images. Scale bar:10 µm (B) Chick cortical neurons, imaged at 12 *d.i.v*., 0.1 Hz, show acidic lysosomes. Boxed region showed all lysosomes were acidic, bar individual alkaline *puncta*, examined in **C**. Scale bar:10 µm (C) Zoomed in individual alkaline organelles found in neuronal processes. Alkaline *puncta* are not enriched for mCherry, imaged at 0.1 Hz. Scale bar: 2 µm (D) ARL8B transfection caused striking alkalinization of lysosomes in HEK293T cells. *Upper panel*: control cells. *Lower panel*: ARL8B-transfected cells. *Inset*: cell protrusions and pH calculation in cell protrusions. Quantification of pH at cell center and cell protrusions in both conditions N = 3 cells each. pH of lysosomes in control cells was unchanged between cell center and cell periphery (both regions pH 4.2 ± 0.1 Average ± StDev), whereas overexpression of ARL8B alkalinized lysosomes at cell center (pH 5.7 ± 0.1) and more strongly alkalinized those at the cell periphery (pH 6.6 ± 0.2). ** = P < 0.001; * = P < 0.018; t-test. Scale bar: 10 µm

Given that we saw no change in lysosomal pH in the tips of migrating cells, we investigated whether localization or direction of lysosomal transport had any noticeable correlation with lysosomal pH. We found that in HEK293T, HeLa and Cos7 cells, there was no significant difference between the pH of peripherally or centrally located lysosomes (Figure 4A), where central lysosomes are defined as those distributed in the first 50% of the cell area from the nuclear envelope to the cell periphery (Figure 4A, single-plane image and pH calculation). For the above experiments HEK293T (pH_central_ = 4.4 ± 0.5 pH_peripheral_ = 4.4 ± 0.5, mean ± Stdev, n = 10 cells) HeLa (pH_central_ = 4.4 ± 0.5 pH_peripheral_ = 4.3 ± 0.5, mean ± Stdev, n = 10 cells), Cos7 (pH_central_ = 4.4 ± 0.4 pH_peripheral_ = 4.4 ± 0.4, mean ± Stdev, n = 10 cells), neurons (pH_cell body_ = 4.4 ± 0.4 pH_neurites_ = 4.4 ± 0.4, mean ± Stdev, n = 4 cells), t-test. In 12 *d.i.v*. chick neurons imaged in neurobasal medium (Figure 4B), we were able to see very occasional, transiently alkaline punctae (Figure 4C **and Movie S8**, imaging rate 0.1 Hz). However, the alkaline organelles were consistently significantly dimmer in the mCherry channel than all other surrounding lysosomes, suggesting that they may be transport intermediates or lysosomes fusing to the plasma membrane as previously described [32] (Figure 4C). These alkaline organelles had no consistent direction of travel.

### ARL8B overexpression causes strong alkalinization of lysosomes

Our results indicated no notable change in lysosomal pH relative to their distribution in the cell. Previous studies have shown that movement of mature lysosomes to the cell periphery correlates with the acquisition of an alkaline pH, driven by specific lysosome-associated transport complexes. We thus tested the effects of ARL8B overexpression, a small GTPase that drives lysosomal transport to the cell periphery [12,33–36].

Overexpression of the GTPase ARL8B showed, as reported, a striking alkalinization of lysosomes (Figure 4D), particularly those at the cell periphery, where HEK293T make lysosome-enriched protrusions (*inset*, Figure 4D). Overexpression of ARL8B moved the pH of peripheral lysosomes from pH_peripheral_ 4.2 ± 0.1 (control ± StDev n = 3 cells) to pH_peripheral_ 6.6 ± 0.2 (ARL8B-transfected, n = 3 cells; P < 0.001; t-test).

In line with previous findings [10], lysosomes in ARL8B-overexpressing cells were more alkaline at the periphery vs. the center of the cell (ARL8B overexpressors: pH_central_ 5.7 ± 0.1 at vs. pH_peripheral_ 6.6 ± 0.2, P < 0.018; t-test), whereas there was no difference in lysosomal pH at center and periphery in control cells (pH 4.2 ± 0.1 [average ± StDev] in both measurements). At the cell center, ARL8B-overexpressing cells remained more alkaline (pH_central_ 5.7 ± 0.1) than controls (pH_central_ 4.2 ± 0.1; P < 0.016, t-test). Our results would indicate that ARL8B overexpression causes lysosomal alkalinization, which becomes more profound as organelles in ARL8B-expressing cells move to the cell periphery, whereas no similar effect is found in wildtype cells. This suggests that alkalinization is more strongly related to the protein complexes recruited to the lysosome than its subcellular localization *per se*.

### Lysosomal alkalinization is not required for TFEB translocation

**Figure 5.**
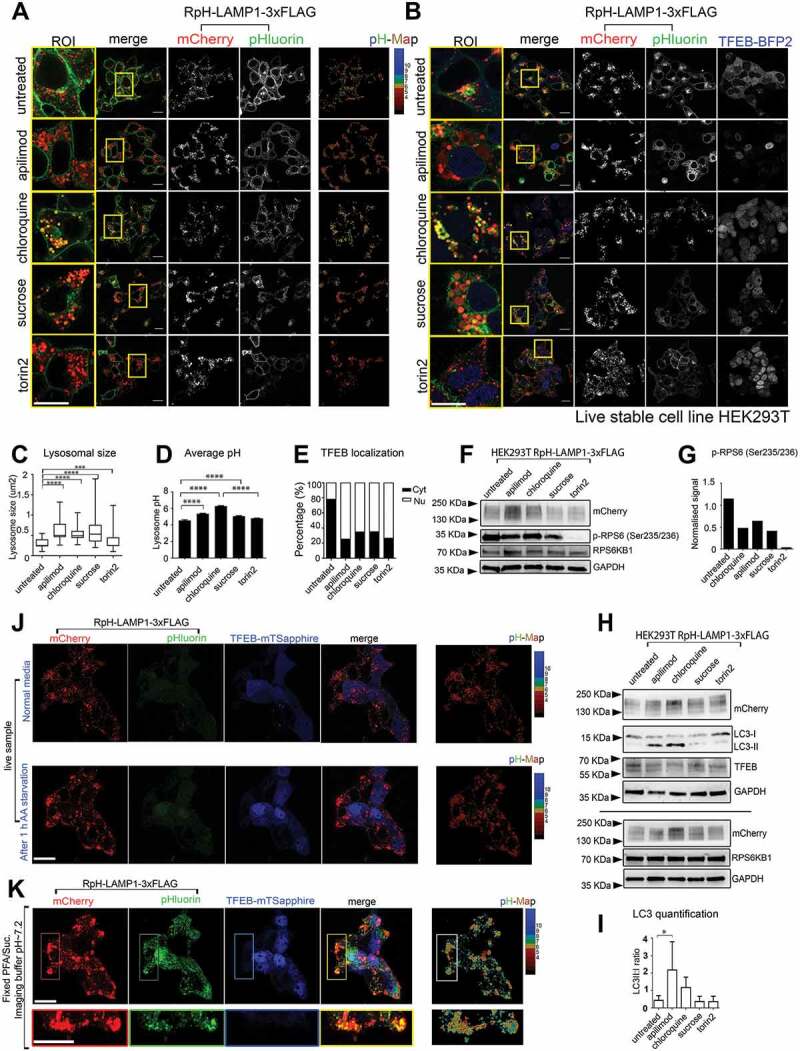
Pharmacological treatment with lysosomal stressors in HEK293T cells. (A) Treatment with apilimod (3 h, 20 nM), chloroquine (5 h, 100 μM), sucrose (4 h, 90 mM), and torin2 (2 h, 250 nM), in stably transduced RpH-LAMP1-3xFLAG cells. Scale bar: 20 μm. (B) Stable expression of mTagBFP2-TFEB showed that the surface pool of RpH-LAMP1-3xFLAG was redirected to lysosomes, and pharmacological intervention caused translocation of TFEB to the nucleus. Scale bar: 20 μm. (C) Quantification of lysosomal size (n = 200–300 lysosomes per condition) (n(fields of view) = 11 untreated; 17 apilimod; 15 chloroquine; 15 sucrose; 16 torin2); statistical analysis: paired t-test. ****: p < 0.0001; ***: p = 0.0001. (D) quantification of average lysosomal pH. (average ± SEM; ****: p < 0.0001; 1 way ANOVA). (E) All treatments caused activation of TFEB nuclear signaling. Untreated n = 19 cells, apilimod n = 19, chloroquine n = 19, sucrose n = 19, torin2 n = 18. (F) Blots on whole-cell lysates from stably transduced RpH-LAMP1-3xFLAG cells visualizing phosphorylation of the MTORC1 target p-RPS6 (Ser235/236) in RpH-LAMP1-3xFLAG-expressing cells. (G) Quantification of p-RPS6 (Ser235/236). (H) Representative blots showing accumulation of autophagic intermediates (LC3 lipidation). (I) Quantification of LC3-II:LC3-I ratio, average ± StDev n = 4 blots 1-way ANOVA, * p < 0.05. (J) Live-imaging of RpH-LAMP1-3xFLAG stable cells transiently expressing TFEB-mTSapphire *Upper panel*: in normal culture medium, *Lower panel*: 1 h after exchange for amino acid starvation medium. See also **Movie S9**. Scale bar:20 µm (K) Fixation and permeabilization of the cells live-imaged in **J**. pH calculation of unquenched lysosomes in right panel. Scale bar:20 µm

We then examined a number of pharmacological treatments known to modify lysosomal signaling and morphology. We chose apilimod [37], an inhibitor of the PIKFYVE lipid kinase, which produces the lysosomal signaling lipid PI(3,5)P_2_ [38,39]; chloroquine (CQ), a weak-base inhibitor of lysosomal function and inducer of macroautophagy/autophagy [40]; sucrose, a lysosomal stressor which induces activation of TFEB-mediated transcription [1] via MTORC1 [41]; and the MTORC1 inhibitor torin2 [42]. Measurements of lysosomal pH after drug treatment in HEK293T stable cells were made for single live imaging planes (n = 19 cells per condition [untreated, apilimod, chloroquine and sucrose] or n = 18 cells [torin2]) (Figure 5A).

Cell lines expressing RpH-LAMP1-3xFLAG were treated in a cell culture incubator with lysosomal stressors in culture medium for the time indicated. Cell culture medium was exchanged with imaging buffer immediately before imaging. Pharmacological treatment of HEK293 T cells with lysosomal stressors yielded varying degrees of lysosomal enlargement and alkalinization (Figure 5A, quantified in Figure 5C–D). The treatment timepoints and concentrations were chosen for the point at which they first exhibited their maximal effects on lysosomal physiology using our probe. When co-expressed with stably expressed mTagBFP2-TFEB (Figure 5B) whose nuclear enrichment is used as a marker of MTORC1 suppression, lysosomes were slightly larger, and the plasma membrane pool of RpH-LAMP1-3xFLAG was reduced (compare pHluorin channel in untreated cells in Figure 5A–B), without any visible translocation of TFEB to the nucleus in control cells. All treatments caused TFEB to translocate to the nucleus, indicating suppression of MTORC1, regardless of lysosomal pH (Figure 5B and quantified in Figure 5E). Treatment with apilimod, chloroquine and sucrose all partially suppressed MTORC1 signaling, assayed by p-RPS6 (Ser235/236) western blot in RpH-LAMP1-3xFLAG cells, and MTORC1 activity was completely suppressed by torin2 treatment (Figure 5F–G).

Quantifying these effects in our cell lines, we saw that the cells with the largest average lysosomal size (Figure 5C) tended toward the most alkaline pH (Figure 5D) in HEK293 T cells. However, there was no strict correlation between average lysosomal size and pH change, and this was also not correlated with the proportion of cells showing nuclear TFEB (Figure 5E). Using these same treatment conditions in Cos7 cells (**Fig. S5A and S5C**), we saw a much more profound change in lysosomal size caused by all treatments (**Fig. S5B**), but only treatment with the weak base chloroquine (CQ) caused lysosomes to become significantly more alkaline (**Fig. S5C**), suggesting that there is a considerable difference in the effects of lysosomal stressors on lysosomal pH in different standard cell culture models. Drug treatments had the expected effect on autophagic signaling in HEK293T, leading to a strong accumulation of LC3-II (blots Figure 5H and quantified as LC3-II:LC3-I ratio in Figure 5I). We also live-imaged RpH-LAMP1-3xFLAG cells transiently expressing TFEB-mTSapphire [43] to determine the response of cells to amino acid starvation. As predicted [9], amino acid starvation caused translocation of TFEB to the nucleus within 30 min, without any loss of lysosomal acidity (Figure 5J **and Movie S9**).

### Examination of purified, functionally intact lysosomes

**Figure 6.**
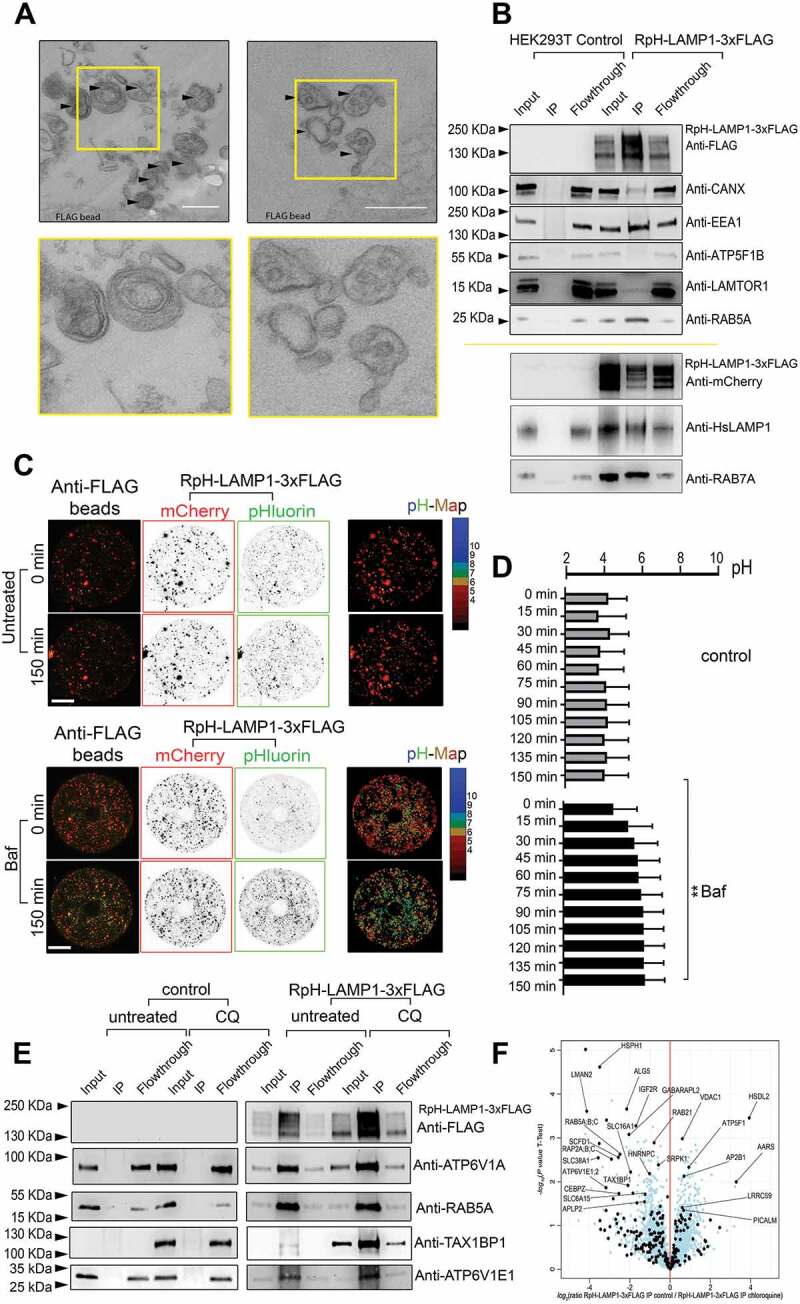
Immunopurification of functional RpH-LAMP1-3xFLAG lysosomes. Stable cell lines expressing RpH-LAMP1-3xFLAG were flash-frozen and cytosolic membranes harvested, pelleted and the pellet fraction immunoprecipitated on FLAG beads. The resulting beads were examined by (A) electron microscopy. Multilamellar and multivesicular organelles indicated by arrowheads (boxed region, inserted lower panel) Scale bar: 1 μm. (B) Bead-purified lysosomes were enriched for markers of the lysosome. (C) Bead-purified lysosomes were acidic and were able to maintain the acidic pH for over 2.5 h. *Upper panel*: untreated beads maintained their pH for over 150 min. *Lower panel*: beads treated with V-ATPase inhibitor bafilomycin A_1_ showed a quick alkalinization of purified organelles. Scale bar:10 µm (D) The pH of purified lysosomes was sensitive to the V-ATPase inhibitor 100 nM bafilomycin A_1_: imaging rate: 1 frame every 15 min. Average ± SEM, t-test at 150 min, ** = P < 0.005. (E) Comparison of immunopurifications from cells not expressing our probe (control) and stably transduced RpH-LAMP1-3xFLAG cells under normal and chloroquine (CQ)-treated conditions. (F) Differential mass spectrometry of chloroquine-treated vs untreated immune-enriched fractions from RpH-LAMP1-3xFLAG HEK293T cells

Our construct also contains a cytosolic 3xFLAG tag, which allows for the purification of lysosomes. To demonstrate the specificity of lysosomal enrichment, we examined bead-purified material by EM, where we saw that the purified membranous organelles were characteristic of lysosomal morphology (Figure 6A). We also made western blots on purified lysosomes, showing that purified fractions were not enriched for markers of mitochondria (ATP5 F1B), while as expected we could detect markers of endosomes (EEA1, RAB5A), lysosomes (LAMTOR1, HsLAMP1, RAB7A) and ER membrane (CANX) (Figure 6B).

To demonstrate that the organelles we precipitated were functionally intact, we made ratiometric measurements of bead-enriched lysosomes showing that purified organelles maintained acidic pH for over 2.5 h under live imaging conditions (Figure 6C, quantified in Figure 6D). This acidic pH was sensitive to the V-ATPase inhibitor bafilomycin A_1_ at a concentration of 100 nM (Figure 6D). This suggests that *ex-vivo* lysosomal pH can be maintained and studied over time courses of at least 2 h. **Fig. S6A** shows a schematic of the lysosomal purification method, and an image of individual anti-FLAG beads decorated with immunopurified lysosomes. Purified lysosomes on beads can maintain their pH in ATP-containing buffer until permeabilized by the ionophore ionomycin (1 μM), whereupon they take on the pH of the imaging buffer, showing that acid lysosomes can maintain a proton gradient once purified by this method. To demonstrate if precipitated lysosomes were compatible with mass spectrometry approaches, we compared lysosomes precipitated from stably transduced HEK293 T in untreated and chloroquine-treated conditions (5 h 100 μM, as in live-imaging experiments above) (Figure 6E–F **and S6, Table S1**), as this treatment had changed both lysosomal size and pH (Figure 5A-D) in this cell line.

The immunoisolated fraction in untreated and chloroquine-treated cells was enriched for lysosomal proteins (see **Table S1 and Fig. S6** for enrichment of proteins and GO-term analyses). Differential mass spectrometry on CQ-treated and untreated immune-enriched lysosomes (Figure 6F) showed a number of proteins to be specifically enriched in purifications from chloroquine-treated cells.

We were able to detect most subunits of the V-ATPase (ATP6V0 [integral V_0_ subunits] A1, D1 and C, and ATP6V1 [soluble V_1_ subunits] A, B2, C1, E1/2, G1 and H) in total cell lysates, all of which were enriched in the immune-enriched lysosomal fraction (**Fig. S6 and Table S1**). Curiously, in chloroquine-treated cells, there was a stronger enrichment of the ATP6V1E1/2 subunit (Figure 6F and western blot against ATP6V1E1 in Figure 6E) than of any other members of the V_1_-ATPase complex. As shown in our mass spectrometry results, other V_1_ subunits of the V-ATPase, such as ATP6V1A, were only mildly enriched in our preparation by treatment with chloroquine (Figure 6E).

Chloroquine treatment stabilized total cellular TAX1BP1 (Figure 6E–F) as previously reported [44] and strongly enriched TAX1BP1 on the lysosomal/autophagosomal fraction (Figure 6E) as well as enriching LC3-like receptors, such as GABARAPL2/GATE-16 (Figure 6F). We also found an increase in IGF2R/CI-MPR (a transcriptional target of nuclear TFEB [1]) and another sugar transporter LMAN2/VIP36 in chloroquine-treated lysosomes (Figure 6F): this may reflect a lack of lysosomal acidity failing to uncouple these receptors from their cargoes to allow its retrieval from nascent lysosomes.

We also found increased enrichment of lysosomal glutamine transporter SLC38A1/SNAT1 [45]. SLC38A1 expression is increased in mutants of the BLOC-1 complex [46], which controls the generation of lysosomal-related organelles, and has been recently found to modulate neuronal MTORC1-RPS6KB1/S6K signaling to stimulate macroautophagy/autophagy [47]. Other lysosomal stressors, such bafilomycin A_1_ treatment, strongly reduces cellular glutamine, suggesting a role of acidic organelles [7] in amino acid flux. Other members of this family, such as SLC38A7/SNAT7 [48] and SLC38A9/SNAT9 [49,50], have been found specifically on lysosomes to mediate amino acid sensing to the MTORC1 complex [51]. Chloroquine treatment also induced an increase in lysosomal SLC6A15/SBAT1, a pH-inhibited branched-chain amino acid (e.g., leucine) transporter, which may suggest roles in metabolism.

## Discussion

We have shown that our probe, harboring a reporter of acidity in the lysosomal glycocalyx, by fusing tandem ratiometric fluorophores with the lysosomal marker LAMP1, allowed robust measurement of lysosomal pH over extended time courses of hours to days in both cell culture and *ex-vivo* immune-enriched lysosomal preparations. This facilitated direct correlation of cell culture images and of purified samples for biochemistry, or *ex-vivo* live-imaged manipulations. The stability and wide applicability of this probe will allow the detailed examination of the complex network that surrounds lysosomal pH homeostasis and to help untangle the controversies in this field due to studies using different conditions, pharmacological treatments and lysosomal labeling methods.

Lysosomal pH and the lysosomal V-ATPase complex is of critical relevance to lysosomal function, since it influences a number of parameters: activation of cathepsins and other lysosomal hydrolases/proteases, as a regulator of lysosome-autophagosome fusion, as a counter-ion for lysosomal chloride channels, and as a driver of amino acid sensing to MTORC1 in the lysosome. Reciprocally, V-ATPase function is regulated itself by lipid composition [52–54], MTORC1 [55], nutrient [56–58] and growth factor [59,60] signaling among others. How this process is regulated, and in what respects lysosomes may differ in proteolytic activity, signaling capacity and physical distribution in the cell, and how much each of these functions are intertwined, is a matter of extensive debate and active research for basic and translational biologists.

The V-ATPase is comprised of two complexes: the membrane-bound V_0_ complex (comprised of V_0_ subunits A to E) and the cytosolic V_1_ catalytic subunit (comprising of the subunits V_1_A-H in the stoichiometry A_3_B_3_C_1_D_1_E_3_F_1_G_3_H_1_) which is responsible for ATP hydrolysis [8]. Proton flux is controlled over short time courses by the regulated assembly and dissociation of the V_1_ complex to the V_0_ complex, and over longer time courses by the trafficking of membrane-associated V_0_-ATPase subunits to the lysosome. The V_1_ complex is recruited to lysosomal V_0_ transmembrane subunits by a wide variety of cellular signals including changes in amino acid levels and alkalinization of the lysosomal lumen [56] to shape proteolysis and lysosomal signaling.

In many circumstances, assembly of the V_1_/V_0_-ATPase complex on lysosomes is considered a proxy for acidification of lysosomes. However, increased V_1_-ATPase recruitment *per se* is insufficient to drive increased acidification, as shown by alkalinizing treatments such as chloroquine (CQ) causing increased V_1_ complex recruitment to the lysosomal fraction [56,61]. In that regard, both V_1_-ATPase recruitment and endpoint assays that measure proteolytic cleavage of model substrates may indicate earlier changes in pH/lytic capacity (as we showed in **Fig. S3A and Movie S2** where a marker of CTSB activity is retained in lysosomes which are neutralized by addition of chloroquine, and our proteomic studies [Figure 6], which showed increased recruitment of V_1_-ATPase subunits to lysosomal fractions under CQ treatment). These probes, as we found, may not accurately reflect the current state of the organelle when measured, but rather the historic activity of that organelle. Therefore, the ability to make continuous measurements of lysosomal pH over a time course is a valuable tool for understanding lysosomal physiology.

Lysosomal pH is of key relevance to both the etiology and treatment of an extensive range of human disorders: lysosomal acidification defects are found in lysosomal storage disorders (LSDs) such as PPT1/CLN1 mutation [62] and nearly all neuronal ceroid lipofuscinoses [63], mitochondrial disorders [64] as well as dementias such as frontotemporal dementia, Parkinson disease [65,66] and Alzheimer disease [67]. Treatments that converge on reacidifying lysosomes have shown promise in whole animal models of LSDs [68] and dementia [69,70], where risk factors for dementias such as the lysosomal size-determinant TMEM106B [5,71] have been found to stabilize the V-ATPase [4] at the lysosome.

pH is also a key modifier of important lysosomal/autophagic signals that feed into indirect MTOR signaling: lysosomal resident channels such as the Ca^2+^ channel TRPM2/melastatin-2 [72], a regulator of autophagy [73] are suppressed by a luminal pH of 6 or higher [74], suggesting pH as a direct regulator of autophagic flux, as also found in recent models of ubiquillin signaling dysfunction [14]. For example, chloroquine treatment reduces lysosomal lytic capacity, presumably by reducing proteolytic activation of cathepsins [75] when lysosomal pH is more alkaline, and concomitantly slows autophagosome-lysosome fusion. This latter function has recently been proposed as the main route of therapeutic action by chloroquine treatment in whole-animal models [76].

In pancreatic cancers, breakdown of albumin through lysosomal hydrolases has been posited as a major nutrient source [77] and that MTORC1-TFEB signaling is increased specifically by acid-selective accumulation of lysosomotrophic drugs, leading to resistance [78]. Likewise, overexpression of the oncogene STAT3 shows that lysosomal STAT3 increases lysosomal acidification [79], overall suggesting a central role in controlling the lysosomal acidification axis for therapy in a wide variety of cancers [80]. Nevertheless, the literature exploring the relationship of V-ATPase function to MTORC1-TFEB signaling presents apparent inconsistencies, likely due to different methods and interpretations of pH measurements over varying pharmacological treatments and cell types. Many treatments which stimulate signaling through the TFEB stress axis are also those which cause defects in lysosomal acidification (Figure 5), although how these mechanisms may work in mammalian cells, and their interaction between each other is not fully resolved. In yeast, for example, genetic conditions that abolish the production of the phosphoinositide lipid PI(3,5)P_2_ lead to a reduction of V-ATPase activity due to reduced V_1_/V_0_ assembly, that can be almost completely restored by the addition of glucose [53], showing that multiple signaling circuits can combine to control lysosomal physiology.

Our own results (Figure 5 **and S5**) showed that drug treatments which change lysosomal pH are largely uncoupled from changes in lysosomal size and changes in MTORC1 signaling (as assayed by TFEB- mTagBFP2 translocation to the nucleus [Figure 5B–E] and p-RPS6 [Ser235/236] western blot [Figure 5H–I]). We found that in stable RpH-LAMP1-3XFLAG lines, that while apilimod, chloroquine and sucrose all caused lysosomal size to increase (Figure 5C), and MTORC1 activity to be reduced (Figure 5B,E-G), chloroquine had a much more significant effect on lysosomal pH (Figure 5D), which could not be explained by an overall block in autophagy, as apilimod-treated cells retained more LC3-II than chloroquine-treated cells (Figure 5H–I). Likewise, more direct treatments, such as MTORC1 inhibition with torin2, completely suppressed p-RPS6 (Ser235/236) signal, promoted TFEB translocation to the nucleus, and made lysosomal size more variable, but had very little effect on LC3 conjugation, or average lysosomal pH.

We also noted that there was some cell-type variance: while lysosomes in chick primary neurons included a small fraction of alkaline organelles (Figure 4B **and Movie S8**), with no observable selectivity of transport direction or location, HeLa and Cos7 did not exhibit many alkaline mCherry-positive structures under our ratiometric imaging conditions using confocal microscopy. We could, however, detect lysosomal fusion events in TIRF in HeLa cells stimulated with thapsigargin, and occasional large fusion events in Cos7 (**Fig. S3**), suggesting that lysosomal fusion is rare, but detectable, in these lines. HEK293 T cells consistently expressed a small fraction of the RpH-LAMP1-3xFLAG probe on the cell surface, which was only visible in the pHluorin channel under our ratiometric imaging conditions, and visible in the mCherry channel if we use non-ratiometric imaging conditions to visualize this minor pool (**Fig. S4**). This surface pool was diminished by all of the lysosomal stressors we used, as well as by overexpression of TFEB-mTagBFP2. We suggest this is because of the increase in the size of the lysosomal compartment provoked by TFEB-mediated transcription. As our probe was expressed under the constitutive *PGK1* promoter, its levels remained unchanged (see mCherry blots in Figure 5F,H **and S5D**) under treatments that encourage transcription of lysosomal proteins. Drug treatments had variable effects depending on the cell line in which they were used: Cos7 cells responded to all of our stressors with a significant change in average lysosomal size, but unlike HEK293 T, chloroquine treatment was the only treatment which had a striking effect on pH in Cos7 (Figure 5 **and S5)**.

In other respects, lysosomal pH was very stable. In all the cell lines we tested, we found no significant difference in the pH of mCherry-positive organelles with subcellular localization (Figure 4A), in migrating or dividing cells (Figure 3, **Movies 6 and 7**), or as isolated and immunopurified organelles on beads (Figure 6C–D), which maintained bafilomycin A_1_-sensitive acid pH for more than 2.5 h.

However, overexpression of lysosomal transport proteins, such as ARL8B, robustly alkalinize lysosomes, particularly those at the periphery (Figure 4). This has been shown by other groups to cause reduced MTORC1 activity [81] and TFEB translocation. We found that in addition to the effect on peripheral lysosomes, ARL8B overexpression made even centrally located lysosomes more alkaline than control central lysosomes (Figure 4D), suggesting that ARL8B overexpression generates alkaline pH, which may become more pronounced as the lysosome matures and moves to the cell periphery. This is consistent with studies in *Drosophila*, which show Arl8 loss causes general acidification of lysosomes in clonal knockout tissues [82].

Other manipulations affecting lysosomal subcellular distribution, such as amino acid depletion, modulates both lysosomal positioning to the cell periphery and MTORC1 responsivity of lysosomes via BORC [11]. Recent studies suggest that amino acid starvation increases V-ATPase assembly and catalytic activity, presumably driving stronger proteolysis and recovery of amino acids. This process could be blocked by chloroquine [56], which in other experiments, including our own, has been shown itself to increase V-ATPase assembly on the lysosomes, showing that there are multiple recruitment signals for V_1_-ATPase assembly allowing for context-dependent recruitment of these complexes to the lysosome. Consistent with this model, we found that amino acid starvation (Figure 5J **and Movie 9**) did not alkalinize lysosomes, while provoking a robust response in terms of TFEB-mTSapphire translocation to the nucleus.

In our experiments in HEK293T cells, 5 h treatment with 100 μM chloroquine caused robust alkalinization of the lysosome (Figure 5), but also caused increased recruitment of V_1_-ATPase subunits, particularly the ATP6V1E1/2 subunits, as previously reported [56,61] (Figure 6), suggesting that the molecular mechanism of this process is still unclear. The ATP6V1E1 subunit is critical for V_1_-V_0_ assembly in cells in both yeast [83] and human patient cells [84]. In both models [84,85], cells exhibit a heterogeneous array of assembled V_1_ subunits and V_1_-V_0_ complexes, suggesting some functional specialization of V_1_-ATPase complexes at a subcellular level. Clinical studies suggest subunit ATP6V1E1 is required to maintain the majority of assembled V_1_-V_0_ complexes in patient cell lines [84], which suggests that despite its peripheral location on the V_1_ subunit, it may mediate important signaling processes. Subunit ATP6V1E1 contacts RAVE complex for glucose-dependent reassembly of V-ATPase complexes that had disassembled in response to glucose deprivation [86]. In our mass spectrometry experiments (Figure 6F **and Table S1**), we detected a significant increase in peptides from ATP6V1E1/2 under CQ treatment, while other V_1_ subunits were unchanged.

Recent years have illustrated the concept that differently functional lysosomes may be spatially distributed in the cell. Overexpression of factors (such as DCTN2/dynamitin [10] and parts of the BORC-ARL8B complex [34]) which relocate lysosomes to the cell periphery, was correlated with a decrease in CTSL activity, suggesting a change in lysosomal activation by pH. However, our results suggest that the change in lysosomal pH and degradative capacity caused by overexpression of such factors may not be explicitly due to transport to the cell periphery [80], as we see in our control cells that lysosomes can move to the cell periphery without undergoing pH changes of a similar scale as provoked by overexpression of ARL8B. Using HEK293T cells, which have extensive pools of peripheral lysosomes, we found no correlation of lysosomal pH with the distance from periphery (Figures 2 and 4), while overexpression of ARL8B, as predicted, showed a striking shift in pH to more alkaline [10,33,35], suggesting that, in these cells at least, peripheral transport of lysosomes can be uncoupled from the alkalinization activity associated with ARL8B-mediated transport.

For these reasons and many others, it would suggest that the ability to consistently and repeatedly measure lysosomal pH over time and to be able to directly correlate *in vivo* lysosomal physiology with *ex-vivo* purified material for physiological studies of isolated and functional organelles will provide a valuable method. Use of these self-consistent probes will be of enormous assistance in untangling the complicated network of lysosomal protein and membrane trafficking, protein recruitment and signaling modulation which allows the lysosome to master cellular survival in conditions of both health and disease.

## Materials and methods

### Reagents

Fractionation buffer (pH 7.4) consisted of 50 mM KCl (Sigma-Aldrich, P9333), 1 mM EGTA (Sigma-Aldrich, E3889), 5 mM MgCl_2_ (Sigma-Aldrich, M8266), 50 mM sucrose (Sigma-Aldrich, S7903), 20 mM HEPES (Sigma-Aldrich, H3375) pH 7.5, 2.5 mM ATP (Sigma-Aldrich, A2383) and protease inhibitors (Roche, 4693159001). Calibration buffers for measurement of lysosomal pH consisted of 140 mM KCl, 1 mM MgCl_2_, 0.2 mM EGTA, 20 mM HEPES for buffers at pH 7.0–8.0, 20 mM MES (Sigma-Aldrich, M3671) for buffers pH 4–6.5.

The following primary antibodies were from Proteintech: RAB5A (11947-1-AP); RPS6KB1/p70 S6K (14485-1-AP); TGOLN2/TGN46 (13573-1-AP). The following primary antibodies were from Abcam: LAMP1 (ab107597, used only for immunofluorescence); LAMP2 (ab25631); GAPDH (ab9485); ATP5 F1B/ATPB (ab14730); ATP6V1A (ab199326); ATP6V1E1 (ab111733); RAB7A (ab137029); GOLGA2/GM130 (ab52649). The following primary antibodies were from Cell Signaling Technologies: EEA1 (C45B10); DYKDDDDK (14793); LAMTOR1 (8975). The anti-RFP primary antibody was from Chromotek (6G6). The anti-HsLAMP1 antibody used for western blot was from BD Biosciences (BD555798). The anti-LC3 primary antibody was from Nanotools (5F10). The anti-CYCS/cytochrome C antibody was from BioLegend (612302). The anti-VPS35 antibody was from Santa Cruz (sc374372). The anti-TFEB and anti-p-RPS6 (Ser233/236) primary antibodies were a generous gift from Prof. Sylvie Urbe and Prof. Michael Clague (Department of Cellular and Molecular Physiology, Institute of Translational Medicine, University of Liverpool, UK). The following secondary antibodies were from Pierce, goat anti-mouse HRP (31430); goat anti-rabbit HRP (31460). The donkey anti-goat HRP secondary antibody was from Abcam (ab6885). The goat anti-mouse and goat anti-rabbit Alexa Fluor 647 secondary antibodies were from Life Technologies (A21236 and A21245, respectively). The following chemicals were from Sigma-Aldrich: bafilomycin A_1_ (B1793); nigericin (N7143); torin2 (SML1224); thapsigargin (T9033), ionomycin (I0634). Apilimod was from Bio Vision (B1129-5). Dextran Alexa Fluor 647 was from Life Technologies (D22914). The ARL8B plasmid [35] was a kind gift of Dr. Mahak Sharma (Department of Biological Sciences, Indian Institute of Science Education and Research‐Mohali [IISERM], India). RpH-LAMP1-3xFLAG was PCR amplified into the gateway pDONR223 vector (discontinued, Invitrogen) using the BP Clonase II enzyme mix (11789100, Invitrogen), prior to recombination into the pLenti PGK Puro DEST gateway lentiviral vector (Addgene, 19068, E. Campeau) using the LR Clonase II enzyme mix (11791100, Invitrogen) to produce pLenti PGK-Puromycin RpH-LAMP1-3xFLAG.

TFEB-mTAGBFP2 was PCR amplified into the gateway pDONR223 vector prior to recombination into the pLenti6.2-ccdB-3xFLAG-V5 gateway lentiviral vector (Addgene, 87071, M. Taipale) to produce pLenti-CMV-Blasticidin TFEB-mTagBFP2. The fluorophore mTSapphire [43] was codon-optimized and synthesized by IDT-DNA, and subcloned into TFEB plasmid to obtain TFEB-mTSapphire.

### Generation of lentiviral stable cell-lines

HEK293T (takarabio, 632180) cells were transfected with pLenti PGK-Puromycin RpH-LAMP1-3xFLAG alongside a lentiviral packaging mix consisting of PLP1, PLP2 and VSVG plasmids (Thermo Fisher Scientific, A43237) using Lipofectamine 2000 reagent (Invitrogen, 11668019). At 24 h post-transfection, the media was collected and used to transduce HEK293T cells with the pLenti PGK-Puromycin RpH-LAMP1-3xFLAG virus. Puromycin (Sigma-Aldrich, P8833, 2 μg/ml) was added to the cells 24 h-post-transduction, and media changed every 48 h following this until individual colonies were visible. Colonies were subsequently expanded to generate clonal stable lentiviral cell lines. HEK293T cells stably expressing RpH-LAMP1-3xFLAG were subsequently transduced with pLenti-CMV-Blasticidin TFEB-mTagBFP2 virus. Blasticidin (Millipore, 203350, 10 μg/ml) was added to cells 24 h-post-transduction, and cell lines generated as described.

### Cell lysis for western blotting

Confluent dishes of HEK293 T cells, HEK293 T cells stably expressing lentiviral RpH-LAMP1-3xFLAG, HeLa cells ± transient transfection with pLenti PGK-Puromycin RpH-LAMP1-3xFLAG and Cos7 cells ± transient transfection with pLenti PGK-Puromycin RpH-LAMP1-3xFLAG plasmid were washed 2 x in ice-cold PBS (Sigma-Aldrich, 11666789001) prior to lysis in RIPA buffer (Sigma-Aldrich, R0278) on ice for 20 min. Cell lysates were centrifuged at 13,500 rpm for 10 min at 4°C and protein concentrations measured prior to analysis.

### Dynabead cross-linking with anti-Flag

Magnetic Dynabeads (Life Technologies, 10004) were incubated with Rabbit anti-FLAG (NEB, 14793S) in PBS-0.01% Tween 20 (Thermo Fisher Scientific, 10485733) rotating for 15 min at room temperature. Dynabeads were then cross-linked with dimethyl pimelimidate (Sigma-Aldrich, D8388) in 0.2 M triethanolamine (Sigma-Aldrich, 90279) pH 8.2 rotating for 30 min at room temperature. The cross-linking reaction was subsequently stopped with 50 mM Tris-HCl (Sigma-Aldrich, T5981) pH 7.5 rotating for 15 min at room temperature.

### Isolation of intact lysosomes through immunoprecipitation

Intact lysosomes were isolated from HEK293T cells stably expressing lentiviral RpH-LAMP1-3xFLAG using Magnetic Dynabeads cross-linked with anti-Flag antibody. Confluent 15 cm dishes were washed 3x in ice-cold PBS then frozen at −80°C in 1.25 ml fractionation buffer for 5 min prior to scraping the cells into a glass dounce homogenizer and lysed using 20 strokes. The nuclear fraction was pelleted at 435 x g for 10 min at 4°C. Cytoplasmic fractions were subsequently centrifuged 17,900 x g for 15 min at 4°C to pellet small organelles. The pellet was resuspended in 0.5 ml PBS supplemented with 2.5 mM ATP and protease inhibitors or PBS supplemented with 2.5 mM ATP, 50 mM sucrose and protease inhibitors (indicated in figure) prior to incubation with anti-FLAG magnetic beads for 2 h at 4°C 10 rpm. Beads were then washed 3x in PBS prior to imaging, analysis by western blotting or mass spectrometry.

### Imaging of intact lysosomes bound to anti-FLAG magnetic beads

Intact lysosomes were isolated from HEK293 T cells stably expressing lentiviral RpH-LAMP1-3xFLAG using anti-FLAG magnetic beads as described above. Lysosome-bound anti-FLAG magnetic beads were imaged on a Leica LSM-800 confocal microscope using 488 nm and 561 nm lasers and 63x oil objective. Z-stack images were collected at room temperature in fractionation buffer at intervals specified in the figure legends.

### Analysis of anti-FLAG magnetic bead-bound lysosomes by western blotting

Following immunoprecipitation, lysosome-bound anti-FLAG magnetic beads were boiled in Laemlli buffer prior to visualization by western blotting. Samples were run on pre-cast 4–20% TGX gels (Bio-Rad, 456–1903), transferred onto nitrocellulose membranes (Sigma, GE10600002), and blocked for 1 h in 5% milk (Marvel, SKU: 5000183932780) at room temperature. The membranes were incubated with primary antibodies (see reagents, above) overnight at 4°C. Following incubation, membranes were then probed with goat anti-rabbit HRP and goat anti-mouse HRP secondary antibodies for 1 h at room temperature prior to visualization using the Bio-Rad ChemiDoc MP imaging system.

### Electron microscopy of lysosome-bound anti-FLAG magnetic beads

Lysosomes were isolated using the immunoprecipitation protocol described above. Following immunoprecipitation, beads were fixed in 2.5% glutaraldehyde (Electron Microscopy Services, 16120) in 0.1 M HEPES pH 7.4 for 1 h at room temperature. Beads were then post-fixed in 1% osmium tetroxide (Electron Microscopy Services, 19110), 1.5% potassium ferrocyanide (Electron Microscopy Services, 26603–01) in 0.1 M cacodylate (Electron Microscopy Services, 11654) for 1 h and *“en bloc”* stained with 0.5% uranyl acetate (Agar Scientific, AGR1260A). Finally, samples were rinsed in dH_2_O, dehydrated with increasing concentrations of ethanol, embedded in Epon (Sigma-Aldrich, 45359–1EA-F) and cured in an oven at 60°C for 48 h. Ultrathin sections (70–90 nm) were obtained using an ultramicrotome (UC7, Leica microsystem, Vienna, Austria), collected, stained with uranyl acetate and Sato’s lead solutions, and observed in a Transmission Electron Microscope (Leo 912AB, Carl Zeiss, Oberkochen, Germany). Digital micrographs were taken with a 2 Kx2K bottom-mounted slow-scan camera (ProScan, Lagerlechfeld, Germany) controlled by the EsivisionPro 3.2 software (Soft Imaging System, Münster, Germany).

### Analysis of anti-FLAG magnetic bead-bound lysosomes by Mass spectrometry

Following immunoprecipitation (3 biological replicates per condition), lysosome-bound anti-FLAG magnetic beads were boiled in Laemlli buffer, centrifuged for 5 min and extracted supernatants were run on an SDS polyacrylamide gel for 5 min. Stained proteins were excised, digested and analyzed by mass spectrometry-based proteomics as described previously [87]. Briefly, IPs were performed in triplicates from untransfected HEK293 cells, stably transduced HEK293 cells expressing RpH-LAMP1-3xFLAG, untransfected control cells treated with chloroquine and stably transduced HEK293 cells expressing RpH-LAMP1-3xFLAG treated with chloroquine. In addition, total cell lysates of untransfected HEK293 T cells were prepared by the filter aided sample preparation method [88]. The resulting MS and MS/MS spectra were analyzed using MaxQuant (version 1.6.0.13, www.maxquant.org/ [89,90], as described previously [91] using its incorporated label-free quantification tool [92]. First, proteins precipitated from RpH-LAMP1-3xFLAG cells were compared to the total proteome of HEK293T cells. Next, IPs from HEK293T cells and HEK293T cells expressing RpH-LAMP1-3xFLAG were compared. Similarly, HEK293T cells and HEK293T cells expressing RpH-LAMP1-3xFLAG, both treated with chloroquine were compared. Finally, the IPS from untreated and chloroquine-treated HEK293T cells expressing RpH-LAMP1-3xFLAG were compared. Only proteins that were specific outliers in the previous two experiments were labeled in these plots. The methods are described in detail in [93]. All calculations and plots were performed with the R software package (www.r-project.org/; RRID:SCR_001905) and the protocol for label-free proteomics [93].

### Stimulation of lysosomal stress treatments

Stable HEK293T and lipofectamine transfected Cos7 lentiviral cell-lines expressing RpH-LAMP1-3xFLAG were seeded onto glass-bottom imaging dishes at the desired density for each experiment. 24 h post-seeding, cells were treated under one of the following conditions: 90 mM sucrose 4 h; 20 nM apilimod 3 h; 100 μM chloroquine 5 h; 250 nM torin2 2 h; 100 nM bafilomycin A1 plus 10 μM nigericin 30 min. Following treatment, cells were immediately imaged at room temperature in imaging buffer (Life Technologies) on a Leica LSM-800 confocal microscope using 488 nm and 561 nm lasers and 63x oil objective.

### Amino acid starvation experiments

Amino acid-free medium (Stratech, D9800-13-USB) was reconstituted from powder and buffered at pH 7.4. Before addition to cells, starvation medium was warmed and normalized 10 min in the incubator. At the microscope, normal culture media was replaced with the warmed medium. HEK293T RpH-LAMP1-3xFLAG cells expressing transfected TFEB-mTSapphire were imaged using on a Zeiss LSM800 in an environmental chamber with 5% CO_2_ at 37°C.

### TIRF microscopy

Observation of HEK293T stable cells and HeLa cells transiently expressing RpH-LAMP1-3xFLAG by total internal reflection fluorescence (TIRFM) microscopy [94]. We have used multicolor total internal reflection fluorescence microscopy (TIRFM). Cells were imaged at 37°C/5% CO_2_ by TIRFM using a Leica DMi8 with Andor Dragonfly inverted microscope fitted with a 60 × 1.45 N.A. TIRFM Dragonfly was controlled by Andor software (Andor Technologies, Belfast, Ireland). The calculated evanescent field depth was ≈ 60–100 nm, automatically set by the microscope.

### Immunocytochemistry

Stable HEK293T lentiviral cell-lines expressing RpH-LAMP1-3xFLAG were fixed in 4% PFA and 4% sucrose in PBS and permeabilized with 0.1% Triton X-100 (Sigma-Aldrich, X100) for 20 min. Samples were blocked in 0.1% BSA (Sigma-Aldrich, A7906) for 1 h at room temperature prior to incubation with primary antibody overnight at 4°C. Samples were then incubated with goat anti-mouse Alexa-Fluor-647 secondary antibodies for 1 h at room temperature. Samples were mounted using ProLong Gold Antifade reagent (Life Technologies, P36930) prior to imaging on Leica LSM-800 confocal microscope using 488 nm, 561 nm and 647 nm lasers and 63x objective. Nuclei were stained using DAPI (Sigma, D9542) (1:2000)

### Dextran lysosomal marker

Stable HEK293T lentiviral cell-line expressing RpH-LAMP1-3xFLAG and untransduced HEK293T cells were seeded as a co-culture onto glass-bottom imaging dishes at the desired density for each experiment. 24 h post-seeding, cells were loaded with 20 μg/ml Alexa Fluor 647-Dextran (Thermo Fisher Scientific, D22914) for 16 h to label all lysosomes at 37°C 5% CO_2_, and chased for 3 h the following day at 37°C 5% CO_2_ prior to live-cell imaging on a Leica LSM-800 confocal microscope using 488 nm, 561 nm and 647 nm lasers and 63x oil objective.

### CTSB activity label

Stable HEK293T lentiviral cell-line expressing RpH-LAMP1-3xFLAG and untransduced HEK293T cells were seeded as a co-culture onto glass-bottom imaging dishes at the desired density for each experiment. 24 h post-seeding, cells were loaded overnight with CATB FAST 680 (Perkin-Elmer, NEV11112, 1 μM) to label all lysosomes at 37°C 5% CO_2_, prior to live-cell imaging.

### Chick neuron preparation and transfection

Dissociated chick mixed hippocampal-cortex neurons were prepared from brain of chick embryos (E8) similarly as described previously for mouse neurons preparation [95] and following surgery and cell preparation according to published methods [96]. Briefly: we used fertilized eggs from white leghorn chicken (*Gallus domesticus*). The eggs were incubated in the laboratory using an egg incubator (Rcom MARU Deluxe MAX) for 8 d at 45% humidity, 37 ± 1°C. Mixed hippocampi-cortex were isolated from eggs (E8). First, the chorioallantoic membrane was gently cut. The embryo was extracted and the head was isolated. The developing calvarium was removed, followed by the separation of the two hemispheres from the rest of the brain, and finally, the meninges were completely peeled off. The clean hemispheres are fragmented, dispersed mechanically and seeded in culture dishes at 0.4 × 10^6^ cells/ml density. The imaging dishes were pre-treated with poly-D-lysine (Sigma-Aldrich, A-003-M, 0.1 mg/ml). The cells were cultured in neurobasal medium (Gibco, 21103049) supplemented with 2% B-27 supplement (Gibco, 17504044) and 0.5% fetal bovine serum (Invitrogen, 10270106). Transfection was performed using electroporation at the time of dissociation using an Amaxa Nucleofector system, following the manufacturer’s instructions that suggest the use of program O-003 for chicks. The average transfection efficiency was from 40 to 60%, and the majority of transfected neurons showed plasmid expression from day 3–5 until around 14 d in culture.

### Measurement of lysosomal pH, dynamics and size

Lysosomal pH measurements were performed on cells and isolated lysosomes on beads through ratiometric imaging of the mCherry and pHluorin fluorophores. mCherry and pHluorin fluorophores were excited at 561 nm and 488 nm, respectively and both channels acquired simultaneously to minimize misalignment between channels. Calibration curves were performed on both fixed and permeabilized cells and on live, bafilomycin A1-treated and nigericin-permeabilized cells by incubating cells with calibration solutions ranging from pH 4–8 as previously described [97]. Images were analyzed as described in the graphical workflow in **Fig. S1 C**. For live-cell imaging, calibration solutions were supplemented with 100 nM bafilomycin A_1_ and 10 μM nigericin. Ratiometric calculations were performed using Matlab.

### Measurement of central and peripheral lysosome pH

The outer boundaries of HEK293T cells stably expressing RpH-LAMP1-3xFLAG were determined using the pHluorin channel fluorescence at the cell surface. Central/peripheral boundaries drawn by hand in ImageJ (NIH, Public Domain license) at the half-way point between the nucleus and cell membrane. pH was analyzed in Matlab as described.

### Ratiometric calculations and standard curve

Raw fluorescence data in nigericin plus bafilomycin A1-treated cells was acquired for each cell line, in calibrated imaging buffers as previously described [98]. Non-linear fitting of average pixel intensities per object was done according to a standard Boltzmann equation [99–101] shown in Eq1 below, as previously used for cytoplasmic EGFP-mRFP tandem [99] and lysosomal chemical fluorophores [102]:

(0.1) $$R\{PH/CH\}(pH)=R\min+\frac{R\max-R\min}{1+e^{\frac{pka-pH}{Slope}}}$$

Where R is the ratio at the variation of pH, and R_max_ and R_min_ are maximum and minimum ratio range obtainable at either alkaline or acidic pH. To measure pKa of our probe in cell lines, we solved (1.2) below, fitting the raw data from our standardized curves. Once pKa was established, this equation was used to calculate pH from the raw ratiometric data acquired.

(0.2) $$pH(R\{PH/CH\})=pKa-Slope*\ln(-\frac{R\max-R\{PH/CH\}}{R\min-R\{PH/CH\}})$$

### Statistical analyses

For western blotting, all data points represent the mean of at least 2, but often 3 independent biological replicates. Error bars represent standard deviation unless otherwise stated. For images, typically at least 6–8 fields of view were analyzed from at least 3 independent experiments (unless otherwise stated); the data indicate average and the error bars represent the standard error of the mean. For pairwise comparisons, P-values were determined using Student´s t-test, otherwise, one-sided ANOVA was employed.

## Supplementary Material

Supplemental Material

## Software licenses used

Matlab (The MathWorks, Inc.), R software package (www.r-project.org/; RRID:SCR_001905), Mathcad (Mathsoft, PTC), Fijii (imagej.net/Fiji) and Microsoft Office.
