# Supplementary material for: Live imaging of intra-lysosome pH in cell lines and primary neuronal culture using a novel genetically encoded biosensor
Source: Autophagy. 2020 Jun 9;17(6):1500–18. doi: 10.1080/15548627.2020.1771858 (PMC8205096; doi:10.1080/15548627.2020.1771858)
Supplement: Supplemental Material [file KAUP_A_1771858_SM3176.zip › Supplementary information/DropBox link for download.docx]

**Ponsford et al. MOVIES LINK**

<https://www.dropbox.com/sh/8y3ca33yxdcmf7u/AACUuvFDyaizrqSqvTaQXdgTa?dl=0>

Movie1.AVI

Movie2.AVI

Movie3.AVI

Movie4.AVI

Movie5.AVI

Movie6.AVI

Movie7.AVI

Movie8.AVI

Movie9.AVI

**Ponsford et al. TABLE LINK**

**Table S1**:

GO term analysis and Mass spectrometry of enriched proteins in whole cell, membrane and immunoprecipitated membrane fractions.

[**https://www.dropbox.com/s/3xpizec2i9me69a/supplementary%20MS%20data.xlsx?dl=0**](https://www.dropbox.com/s/3xpizec2i9me69a/supplementary%20MS%20data.xlsx?dl=0)
