# Supplementary material for: Live imaging of intra-lysosome pH in cell lines and primary neuronal culture using a novel genetically encoded biosensor
Source: Autophagy. 2020 Jun 9;17(6):1500–18. doi: 10.1080/15548627.2020.1771858 (PMC8205096; doi:10.1080/15548627.2020.1771858)
Supplement: Supplemental Material [file KAUP_A_1771858_SM3176.zip › Supplementary information/Supp_Figures_Movie_Table_Legend R4_CP.docx]

**Figure S1.** Ratiometric imaging with RpH-LAMP1-3xFLAG probe. (**A**) Cartoon of probe design. (**B**) Amino acid sequence and reference to publicly deposited (NCBI) fluorophore sequences. (**C**) workflow showing analysis of pH. Analysis of an image presented in **Fig. 5K**. The red (mCherry) channel was used to generate a binary mask of mCherry-positive objects (lysosomes), which was applied to the original data in the green (pHluorin) and red channels- the ratio of fluorescence in both channels was taken for each pixel and the pH value calculated using the values established from our calibration (see Methods). When returning pH value of organelles, we calculated average pH per object. Scale bar:20 µm.

**Figure S2.** Further calibration of sensor. (**A**) Calibration series in fixed, permeabilized chick primary neurons exposed to a series of buffers at fixed pH. Scale bar:20 µm (**B**) Raw fluorescence intensity in mCherry-positive *puncta*, plot of values recovered from the objects in the cell imaged in **A.** (**C**) Calibration curve for dextran-based sensors. HEK293T cells were labeled overnight with FITC-, Rhodamine B- and Alexa Fluor 647- dextrans, treated with bafilomycin A_1_ and nigericin and exposed to a series of pH-fixed buffers. Experimental trace of fluorescence intensity in each channel (*upper panel*), ratiometric calculation using either Alexa Fluor 647-(blue) or Rhodamine B (red)-dextran as the reference channel *(lower panel*) and raw images of fluorescence in each channel (*right panel*). (**D**) Calculated pH maps in HEK293T labeled with FITC-, rhodamine B- and Alexa Fluor 647- dextrans, using either Rhodamine B or Alexa Fluor 647 as the reference channel (*upper panel*). Calculated pH in RpH-LAMP1-3xFLAG cells labeled with Alexa Fluor 647 dextran overnight and pH calculated using the Alexa Fluor 647 dextran signal as the reference channel (*lower panel*). Scale bar:20 µm (**E**) Quantification of lysosomal pH calculated in D. Lysosomal pH was 4.1 +/- 0.3 (n=50) (FITC/Alexa Fluor 647) and 4.05 +/- 0.3 (n=50)(FITC/rhodamineB), unchanged from RpH-LAMP1-3xFLAG expressing cells 4.09 +/- 0.35 (n=50) (pHluorin/Alexa Fluor 647). n.s= *not significant*, ANOVA. Scale bar:20 µm.

**Figure S3.** Lysosomal function investigated in live cells using the RpH-LAMP1-3xFLAG probe. (**A**) labeling of RpH-LAMP1-3xFLAG stable cell line with CATB FAST 680 (*upper panel*). Treatment with the weak base chloroquine (100 μM) (*lower panel*). Scale bar:20 µm (**B**) pH calculation of images in **A.** Scale bar:20 µm (**C**) Lysosomal exocytosis events were detected in the TIRF field. Upper panel: before thapsigargin, Lower panel: 1 min post addition of 300 nM thapsigargin. Left panel: HeLa cell acquired by spinning disc confocal and middle panel: TIRF channels, Right panel: merged TIRF images. Scale bar:20 µm (**D**) Gallery of a lysosomal fusion event in HeLa detected by TIRF. (**E**) Spontaneous fusion event detected in Cos7 cells, scale bar in inset: Scale bar: 5 μm. (**F**) pH calculation for fusing object in **E.** (**G**) in HEK293T cells a fraction of the RpH-LAMP1-3xFLAG probe was presently unquenched on the plasma membrane. Left panel: spinning disc confocal image, middle panel: TIRF image right panel: merged TIRF images. Scale bar:20 µm

**Figure S4.** Investigation of plasma membrane signal from RpH-LAMP1-3xFLAG probe. (**A**) Stably transduced live RpH-LAMP1-3xFLAG HEK293T cells (*upper panel*) showed a minor compartment of pHluorin fluorescence (asterisks) whose morphology was not preserved by 4% PFA fixation (lower panel). Scale bar: 20 μm (**B**) Imaging of RpH-LAMP1-3xFLAG transduced live HEK293T cells oversaturating the mCherry channel (imaging conditions which do not allow ratiometric calculations). Scale bar: 20 μm. Endogenous HEK293T anti-human LAMP1 immunofluorescence in (**C**) standard (0.1% Triton X-100) and in (**D**) low (0.01% Triton X-100) permeabilization conditions, showed a small fraction of endogenous human LAMP1 is present on the surface of HEK293T cells. Scale bar in C and D: 20 µm

**Figure S5.** Pharmacological treatment with lysosomal stressors in Cos7 cells. (**A**) Cos7 cells transfected with RpH-LAMP1-3xFLAG were left untreated or treated with 3 h 20 nM apilimod; 5 h 100 μM chloroquine; 4 h 90 mM sucrose or 2 h 250 nM torin2. Scale bar: 10 µm Effects of this treatment were quantified for lysosomal size **B** and average lysosomal pH **C**. (**B**) Lysosomal size, 15 cells per condition, n= 245 untreated lysosomes; 138 apilimod; 99 chloroquine; 288 sucrose; 151 torin2 (value +/- StDev , 1-way ANOVA. ***=p<0.001). (**C**) Average lysosomal pH; n= 133 untreated lysosomes; 113 apilimod; 67 chloroquine; 103 sucrose; 303 torin2. 1-way ANOVA. ****=p<0.0001. (**D**) Western blot in HeLa and Cos7 cells treated with lysosomal stressors as above showed no significant difference the abundance of the RpH-LAMP1-3xFLAG probe.

**Figure S6**. Bead purification of RpH-LAMP1-3xFLAG-tagged organelles. (**A**) Schematic diagram of bead preparation protocol. (**B**) Immunopurified lysosomes were acidic. Treatment with the pore-forming drug ionomycin (1 μM) permeabilized immune-enriched lysosomes and caused organelles to assume the pH of the surrounding imaging buffer, unquenching pHluorin in these organelles. *Inset*: dic image of beads. Scale bar: 10 µm **C-E.** Protein enrichment by differential mass spectrometry. For protein identities and GO term analysis refer to **Table S1**. (**C**) Immunoprecipitated RpH-LAMP1-3XFLAG anti-FLAG IP vs whole-cell lysates of the same RpH-LAMP1-3XFLAG transduced cell line to determine proteins enriched on purified lysosomes. (**D**) Anti-FLAG IP of membrane fraction from RpH-LAMP1-3XFLAG anti-FLAG IP vs anti-FLAG IP of membrane fraction of untransduced cell line to control for contaminants caused by the immunopurification procedure. (**E**) Anti-FLAG IP of membrane fraction from chloroquine (CQ)-treated RpH-LAMP1-3XFLAG anti-FLAG IP vs anti-FLAG IP of membrane fraction of CQ-treated untransduced cell line to determine lysosomal proteins enriched under CQ treatment. For comparison of lysosomal fractions under control and CQ-treated conditions, see **Fig. 6E** **and** 6**F**. (**F**) Addition of 50 mM sucrose to the lysosomal purification buffer did not change the amount of purified RpH-LAMP1-3XFLAG (mCherry), soluble V-ATPase subunits (ATP6V1A) or endosomal proteins (RAB5A). Triplicate immunopurifications (+/- sucrose) in RpH-LAMP1-3XFLAG cells and in control cells.

**Movie S1**. Calibration of lysosomal pH in a HEK293T stably expressing RpH-LAMP1-3xFLAG cells in baf plus nigericin-treated cells. Raw data, each individual channel and calculated pH are shown for the time course.

**Movie S2**. Live imaging of RpH-LAMP1-3xFLAG cells (red and green) for 300 min (1 stack every 15 min) after overnight labeling with a substrate (CATB FAST 680), which fluoresces in infrared after CTSB cleavage (blue) in HEK293T RpH-LAMP1-3xFLAG cells (red and green). Treatment with the weak base chloroquine (100 μM) (added at frame 1) neutralized lysosomes (increasing green fluorescence), but the cleaved CATSB substrate was retained in newly alkaline organelles.

**Movie S3**. TIRF imaging in HeLa cells showed lysosomal fusion events (increase in pHluorin channel) triggered by addition of 300 nM thapsigargin. Note that imaging conditions were not optimized for ratiometric acquisition. Imaging rate: 0.25 Hz.

**Movie S4**. Cos7 showed occasional spontaneous lysosomal fusion events. Imaging rate: 0.25 Hz.

**Movie S5**. TIRF imaging RpH-LAMP1-3xFLAG HEK293T stable cell line showed a fraction of the RpH-LAMP1-3xFLAG probe was found on the plasma membrane in unstimulated conditions. Note that imaging conditions were not optimized for ratiometric acquisition. Imaging rate: 0.25 Hz.

**Movie S6**. Ratiometric pH calculation of a maximum projection of a cell undergoing spontaneous cell division over an imaging period of 24 h. One frame every 15 min. Panels from left to right: merged image, mCherry channel, pHluorin channel, pH calculation.

**Movie S7**. 4D movie of stably transduced HEK293T RpH-LAMP1-3xFLAG cells migrating into the cleared area of a wound healing assay. One frame every 15 min, 24 h total imaging period. Upper panels: maximum projection of fluorescence over Z-stack. Central panel: 4D rendering of fluorescence images, Lower panel: DIC imaging, pH calculation of mCherry-positive puncta in maximum projected Z-stack and overlay of pH on DIC image.

**Movie S8**. pH measurement in 12 *d.i.v*. chick cortical neurons electroporated with RpH-LAMP1-3xFLAG. Occasional alkaline *puncta* were visible, but they were significantly less bright in the mCherry channel than other lysosomes and appeared to fuse to the plasma membrane. Images 0.1 Hz.

**Movie S9**. RpH-LAMP1-3xFLAG cells transiently expressing TFEB-mTSapphire underwent the expected lysosomal signalling response to amino acid starvation, by translocating TFEB to the nucleus without losing lysosomal acidity. One frame every 15 s for 70 min.

**Table S1.** GO term analysis and mass spectrometry of enriched proteins in whole cell, membrane and immunoprecipitated membrane fractions.
